# Non-equilibrium induction of tin in germanium: towards direct bandgap Ge_1−*x*_Sn_*x*_ nanowires

**DOI:** 10.1038/ncomms11405

**Published:** 2016-04-20

**Authors:** Subhajit Biswas, Jessica Doherty, Dzianis Saladukha, Quentin Ramasse, Dipanwita Majumdar, Moneesh Upmanyu, Achintya Singha, Tomasz Ochalski, Michael A. Morris, Justin D. Holmes

**Affiliations:** 1 Materials Chemistry & Analysis Group, Department of Chemistry, Tyndall National Institute, University College Cork, Cork T12 YF78, Ireland; 2 Department of Photonics, Tyndall National Institute, University College Cork, Cork T12 R5CP, Ireland; 3 CAPPA, Cork Institute of Technology, Cork T12 T66T, Ireland; 4 SuperSTEM Laboratory, SciTech Daresbury Campus, Daresbury WA4 4AD, UK; 5 Department of Physics, Bose Institute, Kolkata 700009, India; 6 Group for Simulation and Theory of Atomic-Scale Material Phenomena (stAMP), Department of Mechanical and Industrial Engineering and Department of Bioengineering, Northeastern University, Boston, Massachusetts 02115, USA; 7 AMBER, CRANN, Trinity College Dublin, Dublin D02 R590, Ireland

## Abstract

The development of non-equilibrium group IV nanoscale alloys is critical to achieving new functionalities, such as the formation of a direct bandgap in a conventional indirect bandgap elemental semiconductor. Here, we describe the fabrication of uniform diameter, direct bandgap Ge_1−*x*_Sn_*x*_ alloy nanowires, with a Sn incorporation up to 9.2 at.%, far in excess of the equilibrium solubility of Sn in bulk Ge, through a conventional catalytic bottom-up growth paradigm using noble metal and metal alloy catalysts. Metal alloy catalysts permitted a greater inclusion of Sn in Ge nanowires compared with conventional Au catalysts, when used during vapour–liquid–solid growth. The addition of an annealing step close to the Ge-Sn eutectic temperature (230 °C) during cool-down, further facilitated the excessive dissolution of Sn in the nanowires. Sn was distributed throughout the Ge nanowire lattice with no metallic Sn segregation or precipitation at the surface or within the bulk of the nanowires. The non-equilibrium incorporation of Sn into the Ge nanowires can be understood in terms of a kinetic trapping model for impurity incorporation at the triple-phase boundary during growth.

## Abstract (web summary)

Direct band gap nanostructures compatible with Si-based electronics are actively investigated. Here, Biswas *et al*. incorporate unusually large amounts of tin in germanium nanowires by non-equilibrium kinetic trapping, and optical characterizations suggest that the nanowires exhibit a direct band gap.

## Graphical abstract

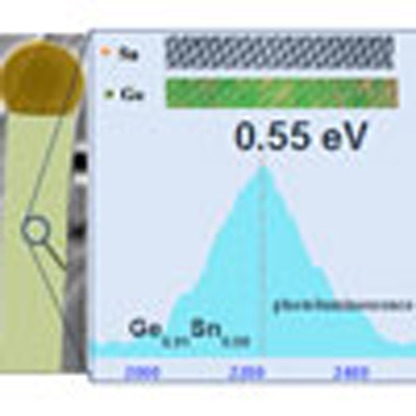

Direct bandgap semiconductor materials are needed for new device architectures such as band-to-band tunnelling tunnel FETs^1^, optical interconnects^2^ and for the development of group IV photonics^3^,^4^ because these technological modules are based on the direct transition of carriers between energy bands. However, a major problem arises with bulk Si and Ge in photonics, optoelectronics and tunnel FETs devices as they are indirect bandgap semiconductors, that is, the lowest-energy transition from the valence to the conduction band involves a change in crystal momentum^5^. Although highly doped, tensile strained Ge results in enhanced direct gap light emission, due to raising of the Fermi level, the doping levels and induced strain required are not practical for many post-CMOS devices. III–V compound semiconductors, for example InP, GaAs, InAs and so on, offer a solution for integrating direct bandgap materials as on-chip photonic and electronic components. However, the monolithic integration of direct bandgap group IV semiconductor materials is expected to lead to lower production costs and higher reliability than hybrid III–V-on-Si approaches^6^. Sn-based group IV alloys are predicted to be tunable direct gap semiconductor materials^7^. Apart from the direct transition of carriers, group IV alloy systems have also been predicted to exhibit high electron and hole mobilities, and low-carrier effective masses, making them ideal material platforms for co-integration of optoelectronic and high-speed electronic devices^7^.

Considering group IV elements, on moving from Si to Ge to Sn, the conduction band at *k*=0 drops in energy until, in grey tin, the material acquires a direct (and vanishing) bandgap at *k*=0 (ref. 5). A direct band system from group IV elements is likely to require the presence of Sn^8^,^9^. Especially for Ge, the small energy separation of 140 meV between the indirect (L) and direct (Γ) conduction band valleys can be overcome by alloying with Sn. Theoretical modelling^10^ as well as photoluminescence experimental studies^11^ have found unstrained Ge_1−*x*_Sn_*x*_ to transition to a direct bandgap material at an alloy composition of no less than 6.5 at.% Sn, although some contradiction regarding this value exists^6^,^12^,^13^, with the range of Sn incorporation to obtain a direct band transition predicted to be between 6.5 and 10 at.%. However, fundamental challenges (low solubility, metallic Sn segregation, lattice mismatch and so on) restrict the growth of Sn-based Si and Ge alloys with a high Sn content (>8 at.%) in any nanoform, for example, thin film, nanowire and so on^14^,^15^.

In recent years, considerable effort has been used to grow Ge_1−*x*_Sn_*x*_ films on Si substrates, where the lattice mismatch with Si is fully relieved by periodic misfit dislocations at the interface with no dislocations into the films^16^. Recent advances in chemical vapour deposition (CVD) techniques have made it possible to grow binary Ge_1−*x*_Sn_*x*_ and ternary Si_*x*_Ge_*y*_Sn_1*−x−y*_ group IV thin film semiconductor alloys using low-temperature epitaxy^17^,^18^,^19^. However, minimal effort has been applied to fabricate group IV direct bandgap materials in one-dimensional (1D) nanoform to keep on track with the miniaturization of Si-based nanoelectronics and to take advantage of their 1D geometry for new age field-effect transistor (FET) devices (finFET, gate-all-around FET and so on). Top-down processing to fabricate good quality (single crystalline, straight, uniform diameter nanowire with no Sn segregation) Ge_1−*x*_Sn_*x*_ nanowires is limited due to under-developed surface and etch chemistries, although encouraging results (with∼8 at.% Sn incorporation) were recently reported on the fabrication of suspended GeSn nanowires through competitive etching between Ge_1−*x*_Sn_*x*_ and Ge layers^20^,^21^. Using bottom-up growth paradigms, Ge and GeSn nanowires were synthesized by utilizing low-melting point Sn metal catalysts, but these techniques produced nanowires either with insufficient Sn incorporation^22^ or low quality (bending and kinking) crystals with non-significant luminescence^23^.

On the basis of thermodynamic limitations, a non-equilibrium growth scenario influenced by the kinetics of the system is required to incorporate a sufficient amount, far from equilibrium, of Sn into a 1D Ge lattice to achieve a direct bandgap transition. The triple-phase boundary at the catalyst-nanowire interface in a bottom-up growth process is known to be a feasible pathway for impurity incorporation in a 1D lattice and can act as a localized non-equilibrium centre for excessive impurity dissolution^24^,^25^. A kinetics-dependent framework was predicted to be responsible for the extraordinary incorporation of impurity adatoms from the catalyst tip.

Here, we report the application of a three-phase bottom-up growth protocol to fabricate highly crystalline, uniform diameter, direct bandgap Ge_1−*x*_Sn_*x*_ nanowires with considerable (*x*>0.09) Sn incorporation; around 10 times the equilibrium solubility. Third-party metal catalysts (Au or AuAg alloy) were used to guide the non-equilibrium incorporation of Sn adatoms into the precipitated Ge bi-layers, where the impurity Sn atoms become trapped with the deposition of successive layers, thus giving an extraordinary Sn content in the alloy nanowires.

## Results

### Growth of group IV alloy nanowires

Participation of Au and AuAg alloy seeds in the bottom-up growth of Ge nanowires has been well-documented by our group in previous reports^26^,^27^,^28^. Similarly, for the growth of Ge_1−*x*_Sn_*x*_ nanowires, we have used dodecanethiol-stabilized phase pure Au and Au_0.90_Ag_0.10_ alloy nanoparticles^29^. These small colloidal alloy nanoparticles were deposited onto silicon (001) substrates (with native oxide) and dried at 180 °C under vacuum, leading to the desorption of the surfactant molecules from the surface of the particles^30^. A liquid-injection CVD technique, using toluene as the solvent phase, was adopted for growing the Ge_1−*x*_Sn_*x*_ nanowires at 440 °C on the surface of Si(001) substrates. Diphenylgermane was used as the Ge source, whereas allyltributylstannane was used as the tin precursor. Similar decomposition kinetics and solubility of the tin and germanium precursors provoked the choice of diphenylgermane and allyltributylstannane, where the Sn precursor has a slightly higher boiling point (360 °C at atm. pressure) than the Ge precursor (325 °C at atm. pressure). The choice of Au and AuAg catalysts and the growth temperature was driven by the Au-Ge and Au-Ag-Ge phase diagrams^30^, where a faster growth rate of Ge nanowires is expected using AuAg catalysts^27^. A faster Ge growth rate provides the opportunity to incorporate more Sn into the Ge lattice, with less chance of segregating on the surface or within the bulk^31^. At our growth temperature (440 °C) Au-Sn or Ag-Sn phase diagrams predict the formation of eutectic liquid alloys (Au-Sn-Ge or AuAg-Sn-Ge) with enormous Sn intakes in the catalyst, without any window for the precipitation of Sn layers^32^.

**Figure 1:**
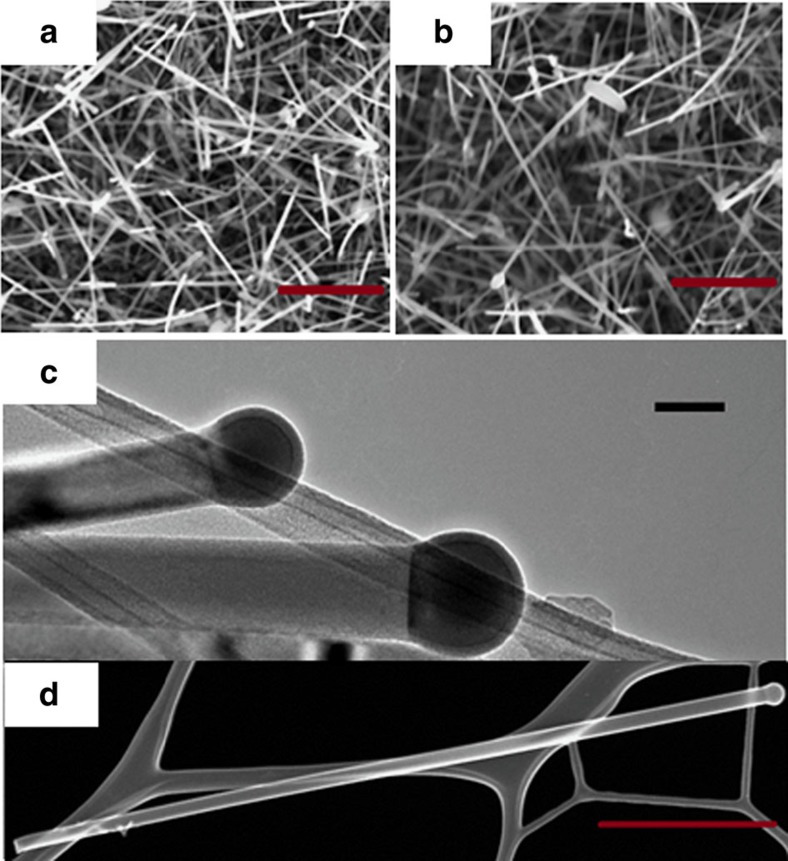
Morphological examination of alloy nanowires by electron microscopy. SEM images of catalysed Ge_1−*x*_Sn_*x*_ nanowires grown using 15 at.% of Sn containing solution with: (**a**) Au (**b**) Au_0.90_Ag_0.10_ catalysts (scale bar, 1 μm). TEM image in **c** confirms the participation of VLS growth mechanism with dark-contrast spherical seed at the tip of the nanowire with AuAg catalysts from precursor solution containing 15 at.% Sn. Scale bar, 100 nm. HAADF STEM image in **d** confirms uniform nanowire diameter along the length with negligible tapering. Scale bar, 1 μm.

Both Au and AuAg nanoparticles successfully catalysed the growth of Ge nanowires after a 2 h time period, as determined by scanning electron microscopy (see Fig. 1a,b). The absence (or very little amount) of particulate deposits, as a byproduct, on the nanowire surfaces and within the samples in general, verifies the controlled growth of the nanowires. The grown nanowires were straight without any observed kinks, bends or curling. A Ge and Sn precursor mixture containing 15 at.% Sn was used as the injection solution for the growth of the nanowires shown in Fig. 1a,b. The lengths of the nanowires grown from both Au and AuAg seeds were in the order of 1–3 μm, whereas their diameters were between 30–70 nm; with a mean diameter of 45.3 and 38.5 nm for Au and AuAg-seeded nanowires, respectively. The bright-field transmission electron microscopy (TEM) image in Fig. 1c confirms the participation of catalytic vapour–liquid–solid (VLS) nanowire growth, as the dark-contrasted partially spherical metal seed can be seen at the tip of the nanowire in the image. A thin amorphous shell can also be observed on top of the nanoparticle seed, but the nanowire diameter is determined by the dimension of the metal seed at the tip. A flat interface was observed after growth between the nanoparticle seed and nanowire, without any lateral side facets at the tri-junction. The nanowires were fairly straight, with no or very little indication of tapering from the seed to the end of the nanowire as shown in the dark-field scanning TEM (STEM) image in Fig. 1d. Metal seeds at the nanowire tips were pinned at the interface, where the contact angle between the interface and the edge of the seed was larger than in the growth of Ge nanowires with Au or AuAg seeds^27^,^28^, due to the relatively low surface energy of Sn-rich metal catalyst seeds. Participation of different growth regime with foreign Au and AuAg metal catalysts was confirmed by the fact that nanowires synthesized without any noble metal seeds, that is, self-seeded growth from Sn seeds, resulted in the formation of very short nanowires (200–300 nm in length), with prominent tapering from the seed-nanowire interface to the very end of the nanowire (Supplementary Fig. 1).

### Elemental analysis of alloy nanowires through EDX

As the primary objective of this work was to fabricate direct bandgap Ge_1−*x*_Sn_*x*_ nanowires with a high Sn content, it is essential to explore the quantitative and qualitative incorporation of Sn in the nanowire body. Energy-dispersive x-ray (EDX) analysis and high-resolution electron energy loss spectroscopy (EELS) provide the means to investigate the chemical environment in the bulk of the alloy nanowire and also at the atomic scale. The composition of the nanowires and the distribution of elements within them were estimated through EDX point measurements and elemental mapping by STEM. For nanowires grown from pure Au catalysts, the amount of Sn in the injecting solution was varied from 10–20 at.%, resulting in a gradual increase in the actual Sn concentration in the nanowires. Very low amounts of Sn (mean concentration of 1.5 at.%) was determined in nanowires using an injecting solution of 10 at.% Sn. Increasing the Sn concentration in the injecting solution to 15 and 20 at.% resulted in the incorporation of Sn in the nanowires at levels of 6 and 9 at.%, respectively. These values of Sn concentrations are much higher (almost 6 and 9 times) than the extrapolated bulk equilibrium solid solubility of Sn in Ge^31^. Although a higher assimilation of Sn in the Ge nanowires was achieved with an injection solution of 20 at.% Sn, this high Sn concentration also resulted in homogeneous nucleation of metallic Sn as spherical clusters (Supplementary Fig. 2). Hence, under our reaction conditions, an initial Sn concentration of 15 at.% was determined to be ideal to obtain Ge_1−*x*_Sn_*x*_ nanowires with substantial Sn incorporation and with negligible secondary nucleation of unwanted spherical particulates in the sample. With the aim of including more Sn into the 1D Ge lattice, Au_0.90_Ag_0.10_ alloy nanoparticle catalysts were used as seeds rather than pure Au, as the alloy seeds have been previously shown to favour faster growth kinetics for phase pure Ge nanowire growth^27^. To avoid spherical metallic Sn clusters in the sample, injection solutions with 15 at.% of Sn were used. A slight increase in the Sn incorporation in the Ge nanowires from 6.0 (±0.5) to 6.6 (±0.6) at.% (error bars are defined in Method section) was observed when using AuAg alloy rather than pure Au seeds, respectively. For an accurate estimation of the amount of Sn included in each nanowire sample, EDX point measurements were performed on 50 different nanowires and mean values computed. An example of a point EDX measurement taken of a Ge_1−*x*_Sn_*x*_ nanowire grown from AuAg seed is shown in Supplementary Fig. 3. The distribution of Sn in the alloy nanowires grown with AuAg seeds was uniform along the length and width of the nanowires, without any segregation near the catalyst-nanowire interface or at the nanowire surfaces (Supplementary Figs 3 and 4). Elemental EDX mapping from a AuAg-seeded Ge_1−*x*_Sn_*x*_ nanowire also confirmed uniform Sn distribution in the entire nanowire volume (Supplementary Fig. 5). The uniform axial and radial distribution of Sn and the minimal tapering of our nanowires also rules out the diffusion of Sn through the nanowire sidewalls as a possible incorporation mechanism. The strong incorporation and uniform distribution of Sn atoms confirms the continuous dissolution of Sn atoms throughout the growth process at the seed-nanowire growth interface.

**Figure 2:**
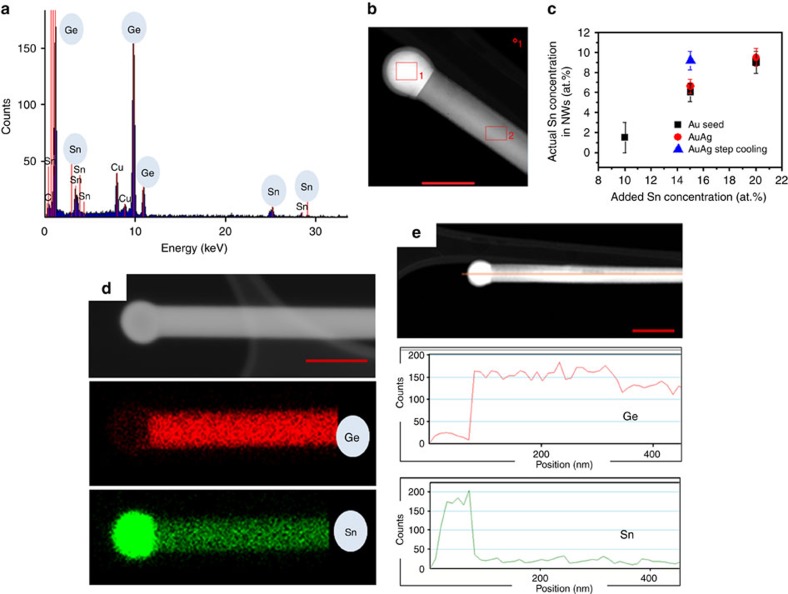
EDX analysis of Ge_1−*x*_Sn_*x*_ nanowires. (**a**) EDX spectrum recorded from the body of an alloy nanowire (selected from the sample with 9.2 at.% average Sn incorporation) showing the presence of both Ge and Sn. This particular nanowire is shown in **b**. Scale bar, 50 nm. Variations in Sn concentration with different catalysts and growth conditions are demonstrated in **c**. Error bar indicates s.d. in EDX measurements measured over 50 nanowires. (**d**) Dark-field HAADF image and EDX mapping for Ge and Sn in a Ge_1−*x*_*Sn*_*x*_ nanowire with 9.4 at.% of Sn. HAADF image with the uniform distribution of Ge and Sn and Sn-rich catalyst is confirmed from EDX mapping and also from HAADF image and EDX linescan in **e**. Red curve denotes linescan for Ge, whereas green curve for Sn. Scale bar, 50 and 100 nm in **d** and **e**, respectively.

To achieve a higher concentration of Sn in the Ge_1−*x*_Sn_*x*_ nanowires, a step cooling method was utilized, where the initial injection of the solution at the growth temperature (440 °C) was followed by an annealing step for 2 h at 230 °C during the cool-down. Motivation to introduce a step cooling at 230 °C was driven by two reasons: a small window in the Sn-rich side of the Au-Sn phase diagram at 230 °C and the bulk Ge-Sn eutectic temperature at around 230 °C. This step cooling technique further forces a colossal amount of Sn (an example is shown in Fig. 2a), with an average concentration of 9.2 (±0.8) at.% (with a AuAg growth promoter and 15 at.% Sn injecting solution), into the nanowire while keeping the nanowire morphology intact (scanning electron microscopy image in Supplementary Fig. 6). The extraordinary amount of Sn incorporation as measured via EDX analysis was also supported through x-ray diffraction measurements (Supplementary Fig. 7). The amount of Sn in the nanowires was calculated as 9.8% from Vegard's law which is an empirical law that relates the substitution of a guest ion into the host lattice with the experimentally observed degree of lattice change. A plot showing the mean Sn concentration in Ge_1−*x*_Sn_*x*_ alloy nanowires as a function of different growth conditions can be seen in Fig. 2b. To confirm the homogeneity of Sn dissolution in the nanowire, that is, to rule out the formation of Sn precipitates or cluster formation in the core or on the surfaces of the nanowires after step annealing, EDX elemental mapping was performed on Ge_1−*x*_Sn_*x*_ nanowires with the highest Sn incorporation, that is, a mean concentration of 9.2 at.%. Elemental mapping of a particular nanowire with a Sn concentration of 9.4 at.% is shown in Fig. 2c. The elemental maps show a homogeneous distribution of Sn in the core of the nanowires without any surface segregation or precipitation near the seed-nanowire interface after step annealing at 230 °C. A high density of Sn was observed at the spherical tips of the nanowires, as confirmed from EDX mapping in Fig. 2c and the linescan in Fig. 2d, confirming the participation of a Sn-rich alloy seed (Sn alloyed with Au or AuAg) in VLS nanowire growth. An elemental EDX linescan of Ge and Sn along the nanowire axis clearly demonstrated the homogeneity of Sn incorporation along the nanowire length even after the step annealing process, thus confirming the continuous dissolution of Sn throughout the nanowire length (part of Fig. 2d). The uniformity of Sn dissolution in the alloy nanowires at the highest average Sn concentration was further confirmed through EDX point scans at different lengths along the nanowires (Supplementary Fig. 8). Even the radial Sn concentration detected by EDX demonstrates (Supplementary Fig. 9 shows the radial line profile for a nanowire selected from the sample with highest Sn content, that is, with step-down cooling) a flat profile (U-shape profile indicates surface segregation), thus again indicating uniform distribution of Sn without any clustering of Sn near nanowire side facets.

### Atomic resolution Sn mapping through EELS

**Figure 3:**
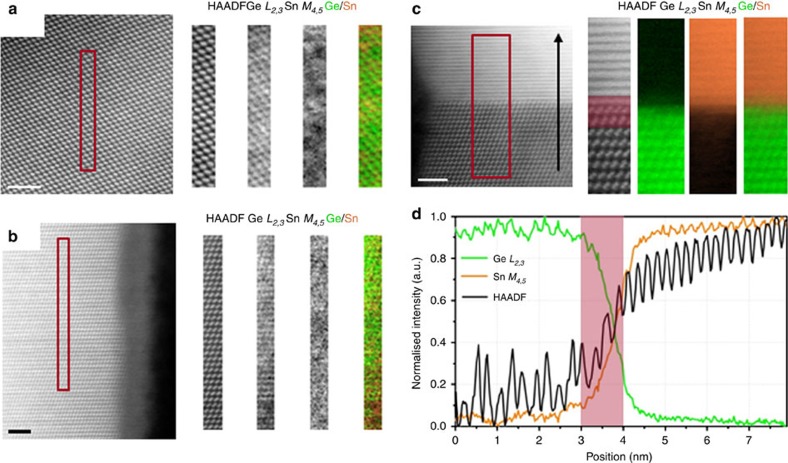
High-resolution EELS mapping of Ge_1−*x*_Sn_*x*_ nanowires. Unprocessed HAADF survey image recorded from the centre (**a**) and near the edge (**b**) of a Ge_1−*x*_Sn_*x*_ nanowire with a Sn incorporation of 9 at.% (area of interest highlighted). Corresponding EELS map for Ge and Sn is also attached along with the simultaneously acquired HAADF image (green: Ge and orange: Sn). For the EELS map, after de-noising by principal components analysis, the background was removed by fitting a power law over a region immediately in front of the core loss edges. The signal was then integrated over a 120 eV window above the onsets of the Sn *M*_*4,5*_ and Ge *L*_*2,3*_ edges. (**c**) HAADF survey image of a seed-nanowire interface region with the Ge and Sn EELS map recorded from the highlighted region. Panel **d** shows a linescan acquired subsequently in the same region. The red shaded area in the linescan corresponds to the same spatial extent indicated on the HAADF image. Scale bar, 2 nm for all the HAADF images.

The uniform distribution of Sn atoms throughout the nanowires also suggests a single-atomic pathway for Sn impurity incorporation. The local distribution of Sn in the nanowires is a fine criterion to determine impurity incorporation and diffusion modes in the nanowires. The possible formation of Sn precipitates in the nanowire bulk or near the nanowire surface suggests multiple impurity incorporation pathways where the impurities are diffused to the preferred lattice sites such as crystal defects. Also, the formation of local metallic Sn segments and Sn–Sn dimers could quench efficient emission from these materials due to the creation of dark trapping sites for charge carriers. To confirm the sparse distribution of Sn in the Ge lattice of the nanowires, we have probed the spatial arrangement of Sn through high-resolution EELS, in a STEM. The spatial arrangement of dissolved Sn in a nanowire sample with the highest Sn incorporation (mean concentration of 9.2 (±0.8) at.%), as determined by EDX measurements were traced (error bars are defined in the Methods section). Two EELS maps were acquired at two vastly different heights along the wires, to make sure Sn was not only present at certain areas of each nanowire, such as close to the catalyst. Maps were de-noised by principal components analysis and the background was removed by fitting a power law over a region immediately in front of the core loss edges. The signal was then integrated over a 120 eV window above the onset of the Sn M_4,5_ and Ge L_2,3_ edges. HAADF images and the corresponding EELS chemical profile recorded from the rectangular box region are depicted in Fig. 3a,b. The HAADF survey image in Fig. 3a was acquired from the bulk of the nanowire to avoid strongly oxidized edges (and the overlap with the Sn edge) and the HAADF image shown in Fig. 3b was acquired closer to the edge of the wire. Both data sets were representative of the whole nanowire. Atomically resolved EELS spectral images highlight the incorporation of Sn in the core of the Ge nanowires. The sparse distribution of Sn in the Ge host lattice is clearly observed in the lattice-resolved EELS maps. No apparent sign of Sn precipitation was detected in the nanowires from EELS mapping, thus confirming the distribution of Sn atoms throughout the Ge_1−*x*_Sn_*x*_ 1D lattice. The Sn EELS M-edge is quite delocalized, thus making it very difficult to resolve Sn as part of the Ge-Sn dumb-bell due to inelastic scattering. A single Sn atom will look blurry and delocalized, especially when the maps are taken over relatively thick regions of the wires as the Sn atom may be buried deep inside the lattice and further scattering will give the impression of a poor image. So the proximity effect of Sn atoms in the lattice may represent as Sn clusters in high-resolution mapping (Fig. 3a,b) with a smaller field of view. Low-resolution EELS mapping from Ge_1−*x*_Sn_*x*_ nanowires also confirmed the sparse distribution of Sn without any formation of metallic Sn hotspots (Supplementary Fig. 10). Hence Sn was distributed uniformly throughout the lengths of the nanowires, but randomly at the atomic scale (as seen in Fig. 2 and Supplementary Fig. 10), without any phase separation. To assess precisely the catalyst-nanowire interface sharpness, EELS chemical maps (recorded from the rectangular red box denoted in the HAADF image attached to the map) and profiles were recorded by moving the electron probe serially across the interface along the line indicated by the black arrow and recording the Ge L_2,3_ and Sn M_4,5_ EELS edges (Fig. 3c). The red shaded area in the linescan (Fig. 3d) corresponds to the same spatial extent indicated on the HAADF image. EELS spectral images for Ge an Sn and chemical line-profiles confirm the very Sn-rich composition of the catalyst seed with sharp composition variation at the seed-nanowire interface. The oscillations of the integrated EELS intensities follow the oscillations of the simultaneously recorded HAADF signal, in both the nanowire and in the seed regions. Random, non-uniform fluctuation of the Sn signal in the line-profiles of the nanowire region also suggests random Ge_1−*x*_Sn_*x*_ alloy formation with high Sn incorporation. The frequency of the HAADF oscillation signal increased in the catalyst thus confirming a much narrower interplanar spacing in the lattice of the catalyst than the nanowire. Abrupt composition fluctuations at the seed-nanowire interface confirmed a continuous trapping and dissolution process for Sn impurity incorporation rather than a Sn layer precipitation and diffusion process.

### Structural characterization of nanowires via STEM and HRTEM

**Figure 4:**
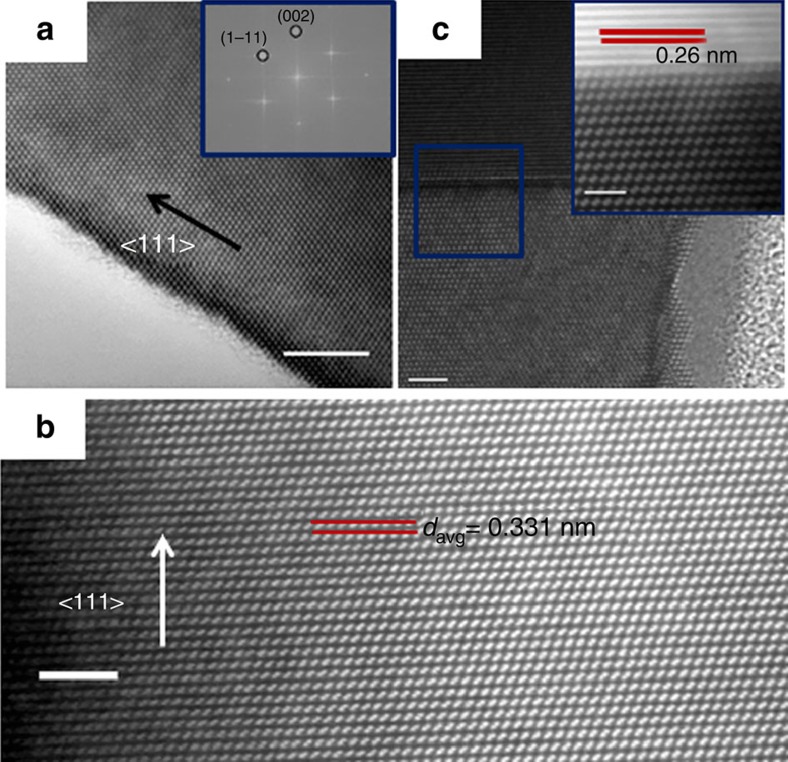
Structural study of alloy nanowires with HRTEM and STEM. (**a**) HRTEM image of highly crystalline Ge_1−*x*_Sn_*x*_ nanowire (9.2 at.% Sn incorporation). Scale bar, 5 nm. FFT pattern in the inset confirms the crystallinity and growth orientation of the alloy nanowire. (**b**) Lattice-resolved STEM HAADF image recorded from the core of the alloy nanowire showing the single crystalline nature with an inter-planer spacing of 0.33 nm. Scale bar, 2 nm. (**c**) High-resolution HAADF image of a seed-nanowire interface (magnified image in the inset) shows abrupt catalyst-nanowire interface with no precipitation of metallic Sn. Scale bar, 2 and 1 nm for the inset.

Impurity atoms (Sn in our case) in nanowires can induce structural defects, such as twins and stacking faults and these defects can act as preferential sites for subsequent impurity accumulation^33^. In other scenarios, pre-formed stacking faults in nanowires due to interface engineering, can also act as preferred sites for the segregation of foreign atoms from catalyst nanoparticles^34^. Hence, it is very important to probe the structural quality of the alloy nanowires to estimate the mode for impurity incorporation in Ge. Also nanowires with defects are not suitable for nanoelectronic devices as stacking faults and twin boundaries can encourage electron scattering^35^. A bright-field high-resolution TEM (HRTEM) image (Fig. 4a) confirms the high crystallinity of a single Ge_1−*x*_Sn_*x*_ nanowire with a 9.2 at.% Sn incorporation and with a 2–3 nm amorphous oxide coating. Fast Fourier transform (FFT) analysis showed a pseudo hexagonal symmetry and the reflections can be assigned to the high-order Laue zone diffraction of {111} and {002} planes in group IV crystals^36^. FFT and HRTEM images depict an interplanar spacing (*d*) between {111} planes in the nanowire to be 0.323 nm, which is very close to the *d* value for bulk diamond Ge crystal (JCPDS 04–0545). An increase in the *d* value from bulk Ge is expected with the incorporation of large amounts of Sn in Ge lattice. However this discrepancy may arise from the fact that this particular nanowire could only be aligned to a relatively minor zone axis. Fig. 4b shows a high-resolution STEM image of another nanowire from the sample with the highest Sn incorporation using the HAADF mode. The image was recorded with <110> zone axis alignment. Generally, the crystal structure of the Ge_1−*x*_Sn_*x*_ alloy nanowires, with various Sn incorporations, exhibited a bulk diamond cubic crystal structure with a 3C lattice arrangement without any stacking faults and twin boundaries, with <111> being the dominant growth direction. Although there is a large lattice mismatch between the components (Ge and Sn) of the alloy^31^, the epitaxial mismatch in the nanowires is compensated by elastic deformation near the hetero-interface and relieved at the nanowire surfaces^37^, thus maintaining highly crystalline nanowires. The liquid eutectic catalyst at the tip of the nanowires can also naturally accommodate elastic strain. Atomic-scale randomness in Sn incorporation in the Ge lattice, as observed through EELS mapping, can generate varied local lattice distortion and spacing at an Ångström scale. To compensate the effect of random alloying on the *d* value, we have calculated the inter planner spacing of 50 successive lattice planes (over >15 nm length) and determined the average *d* value to be 0.331 nm, which is slightly above the bulk 3C-Ge value (Supplementary Fig. 11). Bright-field STEM imaging (Fig. 4c) of the interface between the Ge_1−*x*_Sn_*x*_ nanowires and the metallic tips confirmed the sharp nature of the interface, with no tailing effect or segregation of metal at the interface. For STEM imaging, stacks of images were acquired sequentially at high scanning speed to minimize drift and instabilities and were aligned and summed for a high signal-to-noise ratio. An atomic resolution view of a catalyst-nanowire interface area, indicated by the blue box in Fig. 4c, is depicted in the HAADF image in the inset of Fig. 4c. The sharp contrast in the HAADF intensity at the interface clearly suggests the abrupt nature of the interface. A lattice spacing of 0.26 nm was measured at the metallic tip which is relatively close to metallic Sn (JCPDS cards #04–0673), thus further confirming the formation of a Sn-rich alloy at the tip.

### Raman spectroscopy on alloy nanowires

**Figure 5:**
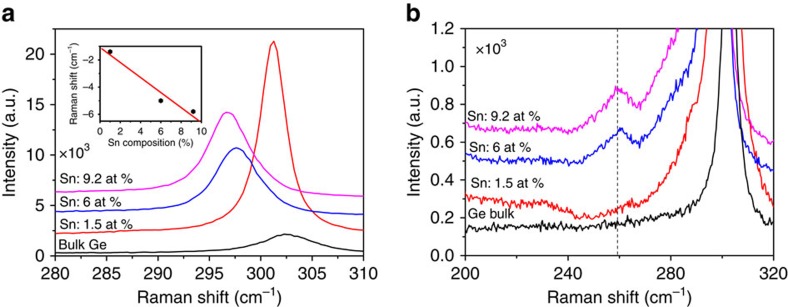
Confirmation of Ge_1−*x*_Sn_*x*_ alloy formation with Raman analysis. Raman spectra of bulk Ge and Ge_1−*x*_Sn_*x*_ nanowires (where *x*=0.06 and 0.092) within the range (**a**) 280–310 cm^−1^ and (**b**) 200–320 cm^−1^. Vertical line in **b** represents position of Ge-Sn vibration. The inset of **a** shows the downshift of Ge-Ge LO mode as a function of Sn percentage. Experimental data are represented with dots which fits (straight line) well with the linear expression, *ω*(*x*)=*ω*_0_+Δ*ωx*.

Raman scattering is an effective tool to estimate the structural and chemical environment in the core of a nanowire. Raman spectroscopy was used to accurately probe the local chemical bonding environment and also to estimate the amount of Sn in the alloy nanowire samples. Figure 5a shows the Raman spectra of alloy nanowires with different Sn concentrations and for reference a spectrum from bulk Ge. The strong peak around 302 cm^−1^ in bulk Ge is attributed to the Ge-Ge LO mode. The Ge-Ge Raman peak progressively shifts to a lower energy with increasing Sn concentration (as determined by EDX measurements). A red shift of 1.2 to 5.9 cm^−1^ of the Ge-Ge LO mode was observed for a variation in the Sn concentration from 1.5 to 9.2 at.%, compared with bulk Ge. We could not compare the Raman shift with a sample of pure Ge nanowires, as the similar growth conditions without any Sn yielded Ge nanowires of entirely different dimensions and morphology^27^. In the alloy nanowires, apart from the Ge-Ge LO peak, additional modes due to Ge-Sn bonds appeared at around 260 cm^−1^, as shown in Fig. 5b. The presence of a Ge-Sn vibrational mode indicates the formation of Ge_1−*x*_Sn_*x*_ alloys, where an increase in the intensity ratio between Ge-Sn and Ge-Ge LO modes with increasing Sn content, implies a larger substitution of Sn in the Ge lattice for Ge_1−*x*_Sn_*x*_ nanowires. The origin of the Ge-Ge frequency shift in the Raman spectra of the Ge_1−*x*_Sn_*x*_ alloys is due to compositional variations and strain effects. Participation of compressive and tensile strain towards the Raman shift is not justified for nanowire samples, as due to the large surface area, strain can be effectively released for these nanostructures. Compositional variations can originate from two factors: (i) mass disorder and (ii) bond distortion. The Ge-Ge LO mode progressively shifts towards a lower frequency with an increasing Sn concentration as displayed in the inset of Fig. 5a. We have fitted the Raman peak shift (Δ*ω*) against Sn composition (*x*), as determined through EDX analysis, with a linear expression, *ω*(*x*)=*ω*_0_+Δ*ωx*, and the obtained value of Δ*ω* was found to be −(64.3±0.1) cm^−1^. This value is consistent with the value of—(68±5) cm^−1^ reported by Li *et al*., who assumed that their alloy films were completely strain free^38^. The linear correlation between the Raman peak shift and the Sn concentration (determined by EDX) in the alloy nanowires further validates the high Sn content in Ge_1−*x*_Sn_*x*_ nanowires. Compared with a few other recent reports, a discrepancy in the Δ*ω* value and Raman shift is observed for the 9.2 at.% Sn containing nanowire sample with a relatively smaller shift in the Ge-Ge LO peak^21^,^39^. This downshift in Raman frequency may arise from the random alloying affect^38^, instead of a spontaneous ordering, as observed in our nanowire sample through high-resolution EELS and TEM measurement.

### Photoluminescence study of Ge_1−*x*_Sn_*x*_ nanowires

**Figure 6:**
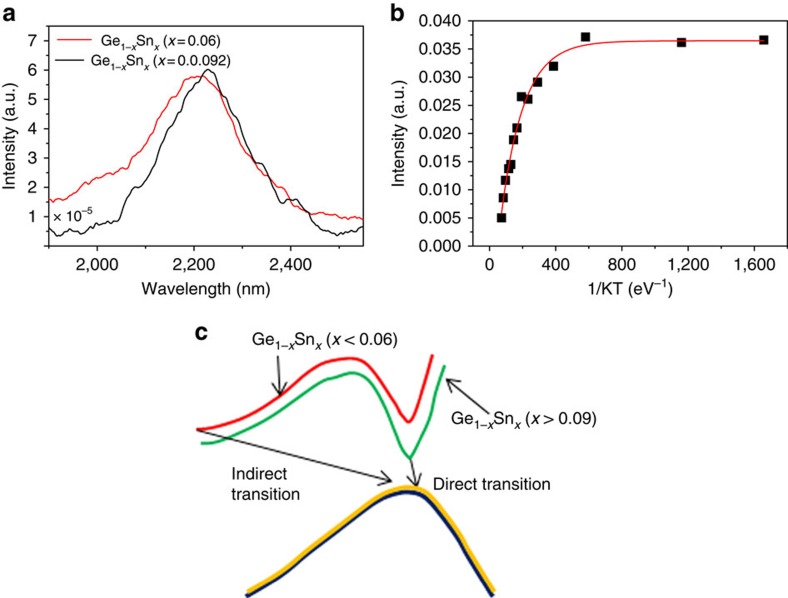
Optical emission characteristics of alloy nanowires. (**a**) Photoluminescence spectra of Ge_1−*x*_Sn_*x*_ nanowires (*x*=0.06 and 0.092) recorded at 7 K. Broadened photoluminescence peak is observed for alloy nanowires with *x*=0.06. Single emission with narrow line-width is observed for Ge_1−*x*_Sn_*x*_ nanowires with *x*=0.092. (**b**) The Arrhenius plot from 9.2% Ge_1−*x*_Sn_*x*_ nanowires in agreement with single exponential decay of photoluminescence intensity with temperature with the coefficient of determination close to unity (*R*^2^=0.986). (**c**) A projection (lines are only guide to eyes) of possible direct and indirect transition pathway for different Sn compositions.

As there are contradictions regarding the amount of Sn needed in Ge_1−*x*_Sn_*x*_ thin films and bulk alloy to obtain a direct bandgap, it is essential to investigate the emission characteristics of strain free bottom-up grown Ge_1−*x*_Sn_*x*_ alloy nanowires. Bandgap information on Ge_1−*x*_Sn_*x*_ alloys was extracted through photoluminescence measurements at low temperature. A photoluminescence study to probe the bandgap characteristics of Ge_1−*x*_Sn_*x*_ nanowire samples was conducted on samples cooled to 7 K, using a He cryostat. Photoluminescence spectra of two nanowire samples (with an average Sn concentration of 6 and 9.2 at.%) recorded at 7 K is shown in Fig. 6a. The photoluminescence spectrum for the nanowire sample with the relatively low Sn content (6 at.%) exhibited a main peak which corresponds to the direct energy gap emission, at a wavelength of around 2,200 nm, with a broad line-width (232 nm) of the emission spectrum. The direct peak is due to the strong radiative recombination of the direct bandgap transition. At this Sn content, separate peaks due to direct and indirect transitions cannot be clearly identified due to the reduced energy difference between the direct and indirect bandgap, resulting in only a single peak with broad line-width (232 nm) and tailing. A large amount of Sn in the Ge_1−*x*_Sn_*x*_ alloy nanowires resulted in the reduction of the bandgap energy difference between the direct and indirect transition, which was approximately 0.14 eV in bulk Ge. Nanowire samples containing a high Sn content (9.2 at.%) exhibited a single photoluminescence emission peak centred at 2,233 nm (band gap (E_g_) around 0.55 eV), with a relatively narrow line-width (202 nm) compared with the photoluminescence plot from the low Sn content Ge_1−*x*_Sn_*x*_ nanowire sample. The relatively narrower line-width of the photoluminescence emission confirms the single energy emission at the Γ point^40^. Typically, a photoluminescence emission with a broad line-width is observed in indirect bandgap alloys with a high Sn content due to the amalgamation of the indirect valley and the direct peak into a single broad emission. However, for our Ge_1−*x*_Sn_*x*_ nanowires, with 9.2 at.% Sn incorporation, the direct band-to-band transition resulted in relatively narrow photoluminescence emission, compared with the broad emission from Ge_1−*x*_Sn_*x*_ alloy nanowires incorporating 6 at.% Sn. A single peak with a relatively narrow line-width could signify emission from only the direct bandgap transition rather than unification of both direct and indirect transitions. The low photoluminescence emission intensity observed from the nanowire samples could be due to the luminescence quenching from metallic Sn impurities, which are present in the catalysts at the tip of the nanowires and also in negligible amounts as spherical particles in samples. Also the high surface-to-volume ratio of nanowires compared with thin films can account for the lower luminescence intensity. Relatively broad photoluminescence spectra for both nanowire samples, compared with previous reports, could result from the random distribution of Sn in the alloys. Sn incorporation in the nanowire samples also showed a standard deviation (around 1%) which may also account for the broadness of the photoluminescence spectra obtained.

The position of the maximum in the direct energy emission from the Ge_1−*x*_Sn_*x*_ nanowires matched well with the reported emission from alloy thin films and disks with similar Sn incorporation (8–10 at.%)^11^,^21^,^40^,^41^. Specifically, the emission energy matched very well with reported data for unstrained GeSn disks^21^. Emission from the Ge_1−*x*_Sn_*x*_ nanowires at low temperature also complements the low temperature photoluminescence observation from GeSn thin films^40^. Thin film samples with 8 and 9 at.% Sn showed similar broad peaks at 10 K, which transformed to a single emission narrow peak with 10 at.% Sn in the film samples^40^. A shift (around 0.01 eV) in the photoluminescence maximum to a lower energy was also observed in the photoluminescence plots of our nanowire samples with an increase in the average Sn concentration from 6 to 9.2 at.%. The shift in the photoluminescence peak energy we observed for nanowire samples incorporating 6 and 9.2 at.% Sn was lower than has been reported for thin film samples^11^,^41^. However, the change in the bandgap of the nanowire alloys with different compositions and morphologies will depend on hole splitting, changes in effective mass, alloy broadening, band-tail states, carrier lifetime and steady state carrier occupation. Also, the different degrees of randomness in the alloys, which may be present between the 9.2 and 6 at.% samples (9.2 at.% samples had an additional step cooling, leading to high degree of randomness as observed in the EELS maps) can affect the band-structure and bandgap tuning. A comparison of photoluminescence spectra at 77 K between pure Ge and Ge_1−*x*_Sn_*x*_ nanowires (9.2 at.% Sn) show a massive red shift in the emission wavelength with the inclusion of Sn (Supplementary Fig. 12). In order to achieve similar emission intensity the excitation power in the case of Ge nanowires was 700 mW in comparison with 30 mW for GeSn nanowire samples. The peak position for the Ge nanowire samples was around 1,750 nm, which matches well with the indirect bandgap of Ge, while for GeSn samples emission was at 2,150 nm.

To predict the direct band transition, a temperature-dependent photoluminescence study between 7 and 160 K was performed. An Arrhenius plot, depicting integrated photoluminescence intensity as a function of inverted temperature is shown in Fig. 6b (for 9.2 at.%) and in Supplementary Fig. 13 (for 6 at.%). The photoluminescence of direct bandgap semiconductors generally decreases in intensity with increasing temperature, which can be attributed to a reduced transfer of electrons from the Γ to L valleys by thermal activation^4^,^42^. Thus the increase in the intensity of the photoluminescence peak with decreasing temperature for GeSn nanowires is attributed to the higher population of the Γ valley. With increasing temperature the fast diffusion of photocarriers toward surfaces and interfaces leads to non-radiative surface and interface recombination, respectively, reducing the radiative transition rate^42^. Furthermore, we also observed broadening of the photoluminescence peak (Supplementary Fig. 12b) with increasing temperature for Ge_1−*x*_Sn_*x*_ nanowires, which can be ascribed to the temperature-dependent broadening of the Fermi distribution of carriers within electron bands^42^. A methodology to discriminate a direct from an indirect fundamental bandgap using temperature-dependent photoluminescence measurements has been presented recently^4^. On the basis of the same arguments, nanowire samples with 6 and 9.2 at.% Sn, manifested by monotonically increasing photoluminescence intensity with decreasing temperature, is similar to photoluminescence observed from direct bandgap III−V alloys or dichalcogenides^43^,^44^. A power-dependent photoluminescence measurement of Ge_1−*x*_Sn_*x*_ nanowires (9.2 at.% Sn) (Supplementary Fig. 12c) depicts the evolution of photoluminescence spectra from the GeSn nanowires under different excitation power densities at 77 K. For the lowest power (*P*_0_=30 mW, enhanced in figure by a factor of 300 for clarity) the photoluminescence peak position was around 2,150 nm. With increasing excitation (up to 16 *P*_0_) spectral broadening was observed, coupled with a blue-shift of the photoluminescence peak position. We attribute this change to carrier filling of closely-spaced Γ and L energy bands. This shift was not observed in the case of Ge nanowires within the scope of available excitation powers between 350 and 700 mW. In addition, in order to achieve similar photoluminescence intensity from both Ge and Ge_1−*x*_Sn_*x*_ nanostructures (Supplementary Fig. 12a), Ge nanowires had to be excited with a laser power of 700 mW, while for Ge_1−*x*_Sn_*x*_ nanowires 30 mW excitation power was sufficient. This enhancement of a factor greater than 20 yields provides further evidence to support the transition from indirect to direct bandgap nanowires with increasing Sn incorporation.

We have calculated the activation energies for non-radiative processes from Arrhenius plots. The activation energy was found to increase with increasing directness of the bandgap^42^. The decrease in the photoluminescence intensity at high temperatures is due to an increase of the non-radiative recombination affects, that is, the activation (deactivation) energy values^42^. Experimental activation energy for 9.2 at.% nanowire samples, calculated from Arrhenius plots, was 7 meV and for 6 at.% nanowire samples was 3 meV. The activation energy value for the 9.2 at.% nanowire samples matches well with the reported value for 12 at.% Sn incorporated thin film samples^42^, which have been designated in previous reports as direct transitions. Furthermore, Arrhenius plots have been fitted with a single exponential function. A coefficient of determination (*R*^2^) close to unity, that is, for a good fit, indicates a single channel of recombination, while poor fit indicates competitive transition channels. For 6 at.% Sn containing nanowire samples the value of *R*^2^ was calculated to be 0.935 (Supplementary Fig. 13), while for the 9.2 at.% Sn incorporated nanowire samples *R*^2^ values of 0.986 were obtained (Fig. 6b). This result indicates that a single charge carrier transition mechanism dominates only for samples with a high Sn content (Fig. 6c). Although the current photoluminescence measurements indicate a direct bandgap, further confirmation regarding the nature of the emission is required, for example, including radiative rates and quantum efficiencies. Spatial orientation, quantum confinement effects and the internal strain in nanowires can strongly impact the electronic band-structure and bandgap bowing^45^ of alloys at the nanoscale.

## Discussion

Ge_1−*x*_Sn_*x*_ nanowires fabricated with Au and AuAg catalysts at 440 °C showed considerable incorporation of Sn in the range around 6–9 at.%, much beyond the bulk equilibrium solubility (around 1%)^45^. The incorporation of Sn in the Ge nanowires through nanowire sidewalls due to homoepitaxy and vapour–solid growth is negligible, as the nanowires were not tapered and demonstrated a uniform radial Sn distribution, as determined by EDX line-profiles (Supplementary Fig. 4). A U-shape line profile with a larger Sn concentration at the edge of the nanowires would have been observed for sidewall Sn incorporation. Size-dependent corrections to the bulk phase diagram due to the influence of capillary forces and stress at the nanoscale^28^,^46^,^47^ results in significant undercooling of the liquid droplet and can in principle alter the equilibrium content of Sn in Ge. Calculations of the nanoscale equilibrium content of a solid impurity in a 1D lattice, taking account of surface anisotropy and elastic stress, do not support a large dissolution of impurity atoms much beyond equilibrium solubility^25^. Careful analysis of the Sn-rich portion of the ternary Au-Ge-Sn phase diagram^48^ shows that for our growth conditions, at equilibrium the Sn-rich (more than 90%) droplet has a Ge:Au ratio of close to unity and the growth should occur via the invariant reaction U4: L+AuSn_2_↔diamond A4+AuSn_4_ mediated by the formation of AuSn intermetallic phases. We did not observe the presence of these intermetallic phases within the nanowire (EDX analysis in Supplementary Fig. 14 shows no traceable amount of Au or Ag in nanowires) or at the hetero-interface through EDX and EELS observations, effectively ruling out equilibrium growth. The droplet morphology/volume also did not change significantly on varying the amount of Sn in the injecting solutions, as would have been expected for near-equilibrium growth^49^. Also, the amount of Sn incorporated in the nanowires increased on using AuAg catalysts which promotes faster Ge nanowire growth kinetics than Au seeds^27^, thus underscoring the role of kinetic factors in the non-equilibrium incorporation of Sn in Ge.

Non-equilibrium induction of Sn impurity in the Ge host is justified through diffusionless solute trapping at a finite growth velocity of the crystals. Solute trapping is a process of solute redistribution at the interface resulting in an increase of chemical potential and deviation of partition coefficient^50^. Local chemical equilibrium at the alloy solidification front at the liquid (seed)–solid (nanowire) interface is relaxed due to a large interface velocity resulting in kinetic interface undercooling. At a high solidification rate at the catalyst-nanowire interface impurity adatoms can be trapped on the high energy sites of the crystal lattice, leading to the formation of metastable solids; for example Ge_1−*x*_Sn_*x*_ with non-equilibrium Sn content, at the nanowire growth front. The kinetic incorporation of Sn is aided by the following factors: (i) Sn diffusion in Ge at the growth conditions is negligible, (ii) the epitaxial mismatch between Sn and Ge results in elastic strains at and near the catalyst-nanowire interface and (iii) the lack of truncating side facets at the catalyst-particle interface. Assuming that growth of the nanowire is layer by layer, the step flow kinetics can result in solute trapping of Sn from the Sn-rich droplet. The deviation of chemical equilibrium at the interface is influenced by the kinetic parameter, that is, interfacial diffusion speed in this case. For bulk metal-semiconductor systems impurity trapping at the liquid–solid interface is highly probable at a very high interface velocity in the order of m sec^−1^ (ref. 51). However, in the 1D Ge_1−*x*_Sn_*x*_ nanoscale systems the growth rate at the liquid–solid interface is only of the order of nm sec^−1^. The growth velocity of Ge_1−*x*_Sn_*x*_ nanowires is much lower (∼0.5–1 nm sec^−1^) than the growth velocity required for kinetic driven solute trapping. However, for our particular system a much higher Sn concentration in the catalyst seed (>90 at.%) than the impurity concentration in a typical bulk solidification process and a continuous Sn flux throughout nanowire growth could account for the high Sn incorporation^34^. In the nanoscale system, where the crystal growth proceeds with the formation of steps at the interface, impurity atoms remains frozen at the step edges on the formation of new row of atoms^25^. Hence, impurity incorporation during nanowire growth depends on the step velocity rather than on the interface velocity. With a high step velocity, the time required for local impurity exchange at the catalyst-nanowire interface decreases thus the rate of solute trapping increases in the nanowire. We delegate a detailed model to a later study, but it is important to note that solute trapping has been implicated in the catalyst incorporation of Al-catalysed growth of Si nanowires^25^. A key difference, although, is that the equilibrium Al-solubility in the Al-Si droplet is much smaller (<5%), unlike the Sn-rich droplet that catalyses the growth of Ge_1−*x*_Sn_*x*_ nanowires. The high Sn concentration around the growing steps is further aided by low Sn diffusivity within the nanowire and along its sidewalls, and the kinetic pathway favors Sn incorporation into the newly forming layer at the expense of elastic strains relative to the pristine Ge crystal. The elastic strains can be effectively accommodated by the Sn-rich droplet. Finally, at equilibrium the catalyst-nanowire interface also involves truncating side facets^52^. These truncating facets are absent in Ge_1−*x*_Sn_*x*_ nanowires and the interface is fully faceted which is indirect confirmation of the elastic strains due to non-equilibrium Sn incorporation. As a result, the Sn incorporation is uniform through the nanowire, as opposed to being localized at the core or within a surface shell.

Induction of Sn in the alloy nanowires is further encouraged with a 2 h annealing at 230 °C, during the cooling down of nanowires (Supplementary Fig. 6). The choice of the step cool-down process and temperature was driven by the existence of a small Sn precipitation window at the lowest eutectic, near the Sn-rich side of the binary bulk Au-Sn phase diagram^32^, at around 215–230 °C. The position and width of the Sn precipitating window in the AuAg-Sn pseudo binary phase diagram was assumed from the Au-Sn and Ag-Sn phase diagrams. This small window encourages further precipitation of Sn from non-equilibrium Sn-rich eutectic Au-Sn and AuAg-Sn catalysts during the annealing process. A deposition and dissolution based process could be responsible for the increase in the Sn amount in the alloy nanowire, where a Sn precipitation from the supersaturated catalyst drop is encouraged at 230 °C. Precipitated tin from the supersaturated catalyst gets further dissolved into the Ge nanowire host lattice at 230 °C due to the eutectic solubility. Metastability and continuous dissolution of Sn in the Ge host is expected at the eutectic temperature. A very large amount of Sn could be dissolved in the Ge lattice in the metastable state as projected in the Ge-Sn phase diagram. So the coincidence of the Sn precipitation from the seed droplet at 230 °C and dissolution of this Sn in the Ge nanowires at the eutectic temperature (at 230 °C) encourages large homogeneous Sn influx into the nanowire. Sn diffusion in Ge at 230 °C is negligible. So a diffusion mediated incorporation process would have a large concentration of Sn near the seed-nanowire interface, with a continuous drop in Sn concentration along the nanowire length. Compositional analysis of 9.2 at.% sample (grown following the step cool-down process) does not demonstrate this trend but shows similar distribution of Sn along the length of nanowires (Supplementary Fig. 8). To confirm the participation of the particular step-down temperature of 230 °C in large Sn incorporation, nanowires were annealed during cool-down at four different temperatures of 210, 220, 230 and 250 °C. However, only those subsequently annealed at 230 °C displayed an increased Sn incorporation, whereas no or negligible increase was observed for other temperatures (Supplementary Fig. 15). So in the step cool-down process, the coincidence of the lowest eutectic in AuAg-Sn system and Ge-Sn system assists to increase the amount of Sn (∼2.5 at.%) further beyond the capability of kinetic trapping. Undercooling and the shift in the liquidus from their bulk counterpart for nanoscale Ge-Sn systems may also be expected for Au-Sn and Ge-Sn binary phase diagram.

In summary, we have successfully fabricated a stable direct bandgap 1D Ge_1−*x*_Sn_*x*_ nanosystem, usually metastable under thermodynamic equilibrium, compatible with existing Si electronics platforms. Kinetic driven solute trapping model in a catalytic bottom-up growth with noble metal catalysts facilitates the dissolution of high amounts of Sn into the alloy nanowires. The sparse spatial distribution of Sn in the nanowires and the formation of GeSn alloys in the nanowire cores were confirmed through atomic resolution EELS mapping and Raman spectroscopy. The use of innovative AuAg alloy catalysts, which enhances nanowire growth kinetics, facilitated larger incorporation of Sn into the Ge_1−*x*_Sn_*x*_ lattice than pure Au catalyst. Calcination of the nanowires near Ge-Sn eutectic temperature during the cool-down further aided Sn precipitation and dissolution to achieve direct bandgap Ge_1−*x*_*Sn*_*x*_ alloy (*x*=0.092) nanowires. Further manipulation of growth temperature, choice of precursors and catalysts could lead towards Ge_1−*x*_*Sn*_*x*_ nanowires with even a higher Sn content. Three-phase bottom-up growth is a feasible way to incorporate large amount of impurities and dopants in semiconductor nanowires. Demonstration of colossal incorporation of foreign atoms in the host semiconductor lattice allows new or added functionalities (strain engineering, controlled defect formation, band-structure modulation and so on) in the existing semiconductor architecture. The demonstrated VLS process with its innovative catalyst and precursors contributes towards the ongoing research on dopant/impurity incorporation in nanowires by directly demonstrating kinetics-dependent tin incorporation in Ge nanowire. The protocol demonstrated here is general and could be applied to other doped or alloy nanowire systems (for example III–V nanowires, ternary group IV alloy nanowires and so on) to create new and innovative nanomaterials for novel physics and devices. This paper also addresses towards the ongoing demand for a nanoscale material for group IV photonics. The demonstration of direct bandgap silicon compatible nanowires will instigate a lot of initiative on fundamental research on band-structure engineering of binary and ternary group IV alloy nanowires and implementation of these nanoscale materials in photonic and electronic devices. The great success of III–V quaternary semiconductors in decoupling strain and band-structure effects suggests that ternary compounds should have a similar impact in the group IV arena. Direct bandgap Ge_1−*x*_Sn_x_ nanowires fabricated through a cheap and feasible bottom-up technique opens up unlimited possibilities in group IV photonics, nanoelectronics and optoelectronics.

## Methods

### Nanowire growth

Continuous-flow reactions for nanowire growth were carried out in a toluene medium using a liquid-injection CVD technique. Metal nanoparticles were spin-coated onto a Si (001) substrate and loaded into a stainless steel micro reactor cell, connected to metal tubing. The catalyst nanoparticle concentration in each case was fixed at 40 μM cm^−3^. Solutions of diphenylgermane and allyltributylstannane in anhydrous toluene were prepared in an N_2_ glove box with a typical Ge precursor concentration of 10 μM ml^−1^ and varying Sn concentrations. The concentration of diphenylgermane in toluene was fixed at 10 μM ml^−1^, whereas tin precursor concentrations were varied from 1–2 μM ml^−1^ for the incorporation of different amounts of Sn in the Ge_1−*x*_Sn_*x*_ nanowires. A precursor solution was loaded into a Hamilton sample-lock syringe inside a nitrogen-filled glovebox. Before injection, the coated Si substrate was annealed for 15 min at 440 °C under a flowing H_2_/Ar atmosphere inside a tube furnace. The precursor solution was then injected into the metal reaction cell using a high-pressure syringe pump at a rate of 0.025 ml min^−1^. A H_2_/Ar flow rate of 0.5 ml min^−1^ was maintained during the entire growth period. A typical nanowire growth time was 2 h. An additional annealing step was also introduced during the cool-down process where the substrate was kept at 230 °C for 2 h under a H_2_/Ar flowing atmosphere. The reaction cell was allowed to cool to room temperature and disassembled to access the growth substrate. Nanowires were washed with dry toluene and dried under N_2_ flow for further characterization.

### Characterization

Bottom-up grown Ge_1−*x*_Sn_*x*_ nanowires were imaged on an FEI Helios NanoLab 600i scanning electron microscope. All EDX measurements were recorded in high-angle annular dark-field mode in the FEI Helios NanoLab 600i operating at 20 kV and 1.4 nA with an attached Oxford X-Max 80 detector. Error in the EDX measurements indicates standard deviation in EDX measurements measured over 50 nanowires. TEM analysis was done in a JEOL JEM-2100 operating at 200 kV in bright-field condition for imaging. High-resolution STEM imaging and EELS mapping was done using Nion UltraSTEM100 microscope, operated at 100 kV. Probe-forming optics were adjusted to deliver a 0.9 Å probe, with 120 pA beam current and 31 mrad convergence semi-angle. EELS data was acquired on a Gatan Enfina spectrometer, at 1 eV per channel to capture both the Sn and Ge edges simultaneously. As a result, the effective energy resolution was limited to 2.5 eV by the detector point spread function (∼3 pixels), even though the cold field emission gun of the instrument had a native energy width of 0.35 eV in the operating conditions. Raman scattering measurements were performed in a backscattering geometry using a micro-Raman setup consisting of a spectrometer (model LabRAM HR, Jobin Yvon) and a Peltier-cooled charge-coupled device detector. An air cooled He-Ne laser of wavelength 633 nm was used as an excitation source. The photoluminescence measurements were performed using a confocal configuration. Samples were cooled to 7 K using a Helium cryostat. A pulsed titanium-sapphire 800 nm laser was used as an excitation source. Laser frequency was 76 MHz and pulse width was 300 fs. The laser beam was focused down to a 50 μm spot and the power was measured to be 500 mW. For temperature- and power-dependent experiments, the structures were encased in a liquid He and liquid nitrogen cryostat, respectively equipped with KBr window and cooled to desired temperature. The photoluminescence emission was collected by a monochromator and then sent to a thermoelectrically cooled, photoconductive, extended-range InGaAs detector, sensitive in the mid-IR spectral range from 1.2 to 2.6 μm and facilitated by CaF2 optical components. Lock-in and chopper was used as a standard noise-cancellation tool.

## Additional information

**How to cite this article:** Biswas, S. *et al*. Non-equilibrium induction of tin in germanium: towards direct bandgap Ge_1−*x*_Sn_*x*_ nanowires. *Nat. Commun.* 7:11405 doi: 10.1038/ncomms11405 (2016).

## Supplementary Material

Supplementary Information Supplementary Figures 1-15
