# Supplementary material for: Non-equilibrium induction of tin in germanium: towards direct bandgap Ge1−xSnx nanowires
Source: Nat Commun. 2016 Apr 20;7:11405. doi: 10.1038/ncomms11405 (PMC4843103; doi:10.1038/ncomms11405)
Supplement: Supplementary Information — Supplementary Figures 1-15 [file ncomms11405-s1.pdf]

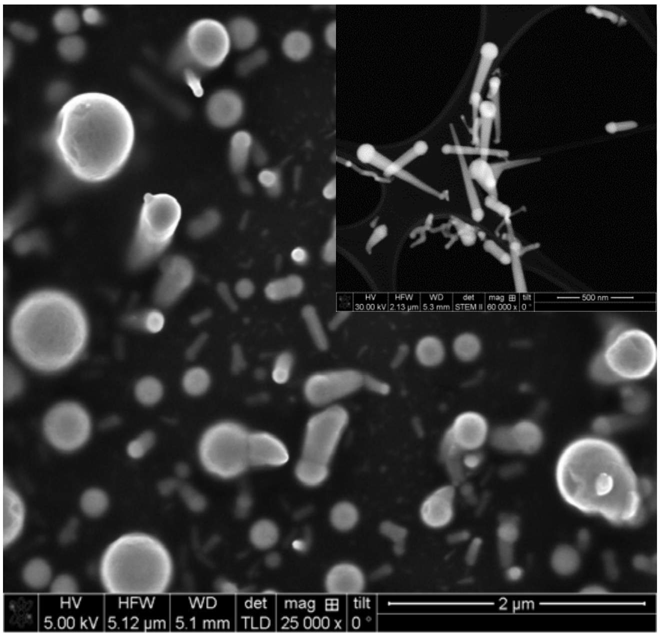

Supplementary Figure 1: **GeSn nanowire with Sn catalyst**. SEM image of the nanowire grown without any foreign metal catalysts (Au or AuAg) shows tapered nanowire with thicker top and thinner bottom with 7.5 µL (15 at.%) of Sn precursor in the injecting solution . Nanowires are much shorter than the Au or AuAg seeded growth after 2 hrs growth time with lower Sn incorporation (~ 3.5 at.%). Dark-field STEM image in inset shows tapered nature of nanowire. Self-seeded growth of $Ge_{1-x}Sn_x$ occurs near equilibrium. The droplet volume expands during nanowire growth as the expansion of Sn catalyst droplet is much faster than the Sn incorporation rate from seed to nanowire. Expansion in the volume of Sn catalyst droplet during growth drives the tapering of nanowires. The combined effect of slow growth kinetics of Ge nanowires from self-catalytic Sn seeds and continuous expansion of the triple-phase interface due to a constant Sn flux encourage prominent tapering in the nanowires without any Au and AuAg growth promoter.

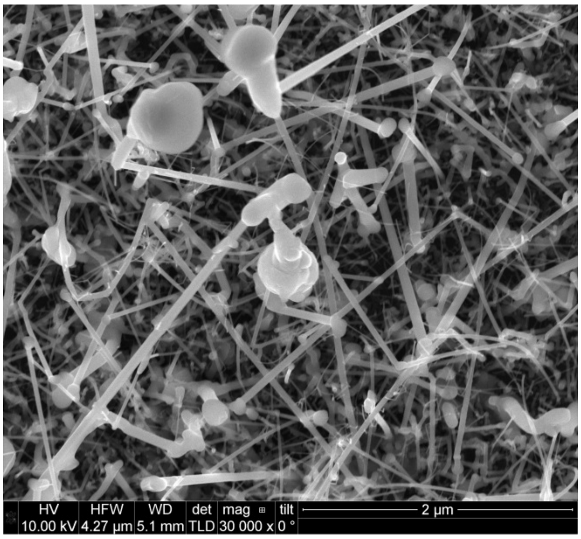

Supplementary Figure 2: **Nanowires grown with high Sn injection.** SEM image of the nanowire grown with Au catalyst shows and with 20 at.% Sn in the injecting solution shows spherical clusters due to homogeneous nucleation of Ge and Sn. Also bimodal distribution in diameter is observed with high Sn in precursor solution.

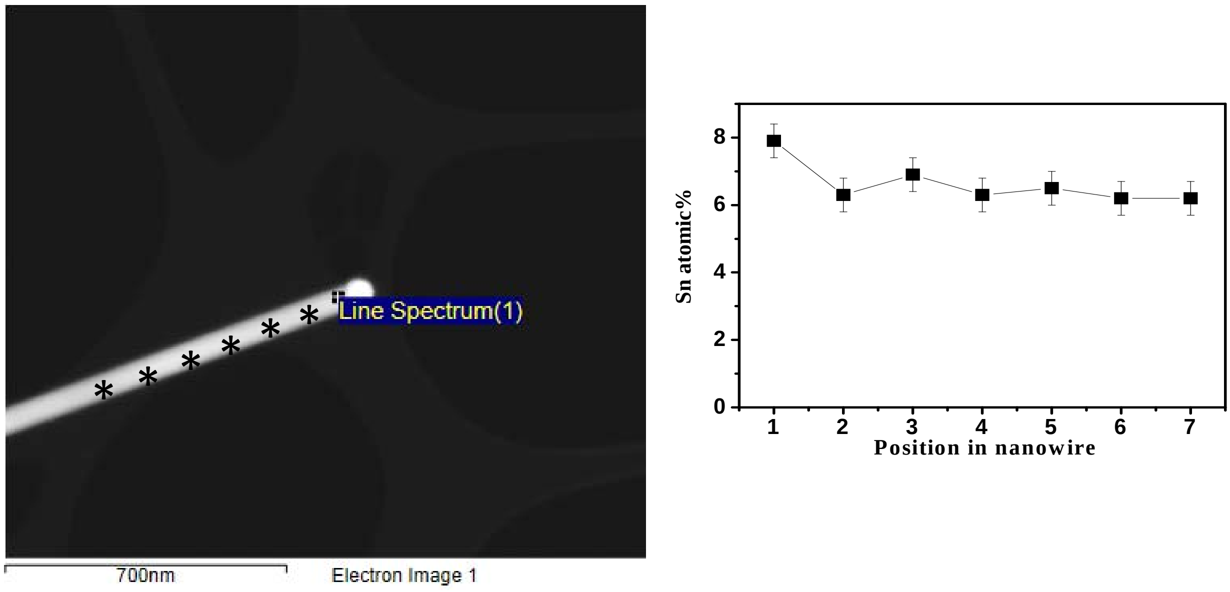

Supplementary Figure 3: **Sn distribution in alloy nanowire with AuAg seed**. EDX scan along the length of a nanowire grown with AuAg seed shows uniformity in Sn distribution throughput the nanowire length. Error bar represent typical error of 0.5 at.% in EDX measurement.

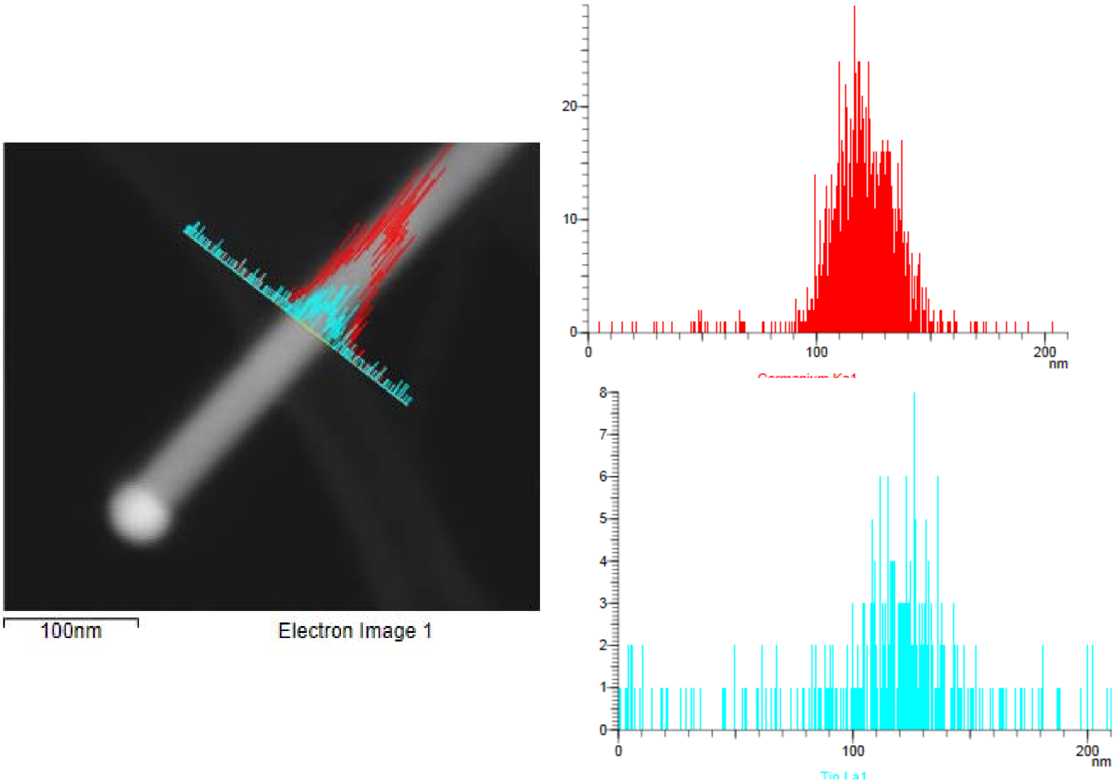

Supplementary Figure 4: **Radial Sn distribution in alloy nanowire with AuAg seed.** EDX radial concentration profile of Ge and Sn from a nanowire with 6.3 at.% of Sn grown with AuAg catalyst. EDX profile shows no segregation of Sn at or near nanowire surface.

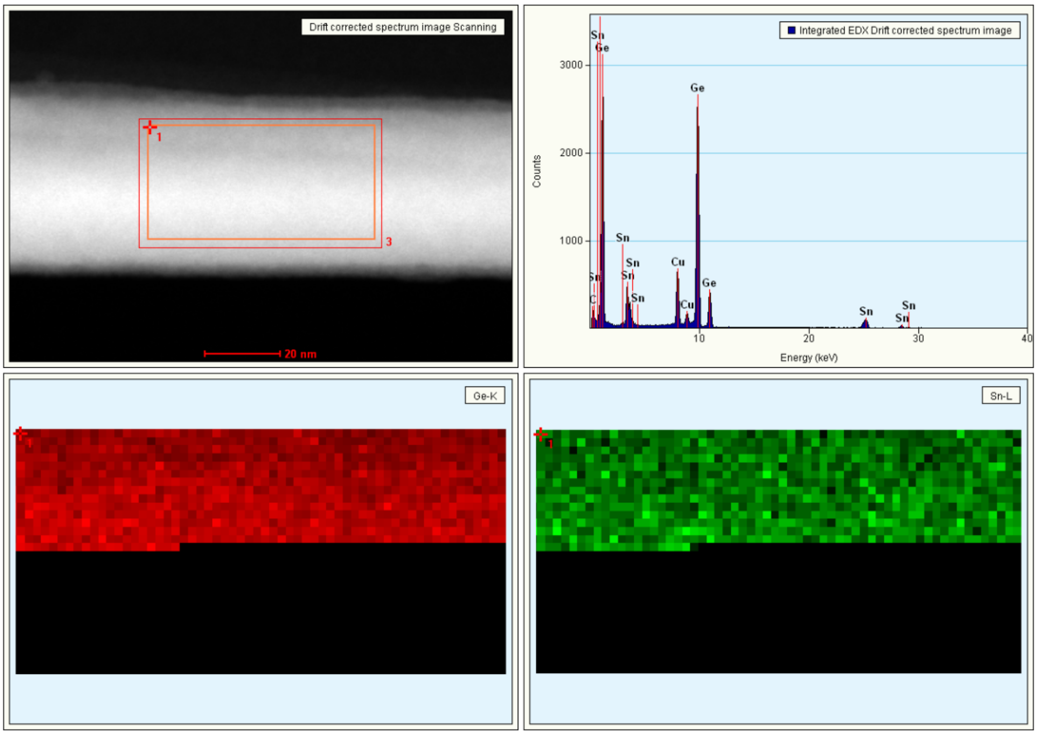

Supplementary Figure 5: **Sn mapping in alloy nanowire with AuAg seed.** EDX mapping from an AuAg seeded $Ge_{1-x}Sn_x$ nanowire body.

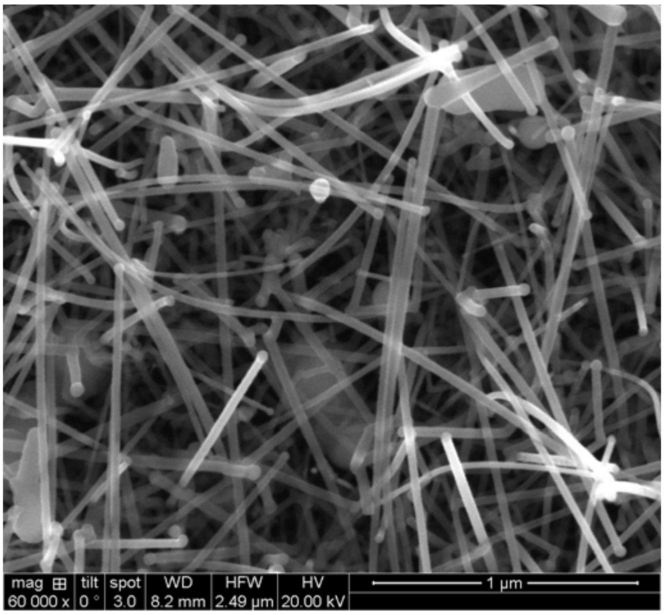

Supplementary Figure 6: **Nanowires grown with a step cooling method**. SEM image of the nanowire grown with AuAg catalyst with 15 at.% Sn in the injecting solution. Nanowires morphology remains intact after a step cool down process at 230 ºC.

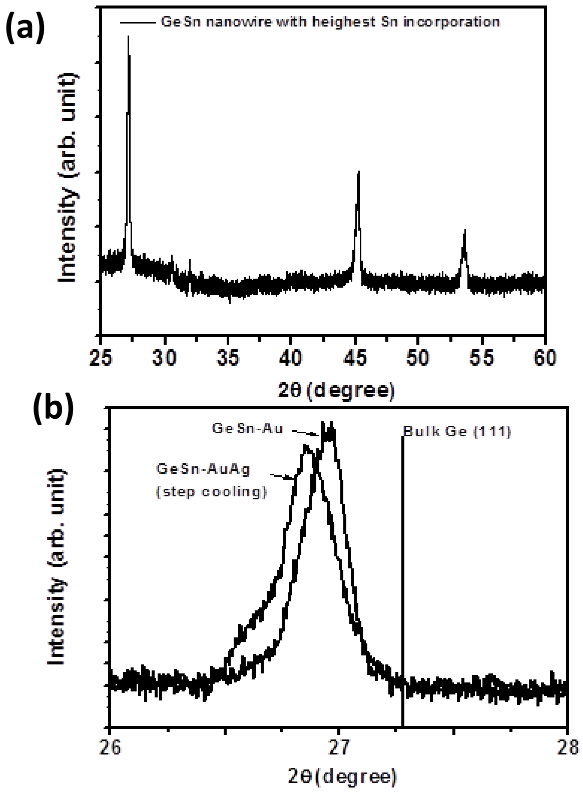

Supplementary Figure 7: **Sn content in alloy nanowires from X-ray diffraction.** (a) X-ray diffraction spectrum from the $Ge_{1-x}Sn_x$ nanowire sample grown with AuAg seed and with an additional step cooling. Lattice constant calculated for this nanowire is 5.739 Å with Sn concentration of 9.8 % according to Vegard's law. (b) X-ray diffraction shows relative shift in the (111) Ge reflection for $Ge_{1-x}Sn_x$ nanowire with AuAg seed (with an additional step cooling) and $Ge_{1-x}Sn_x$ nanowire with Au seed (measured Sn~ 6.8%).

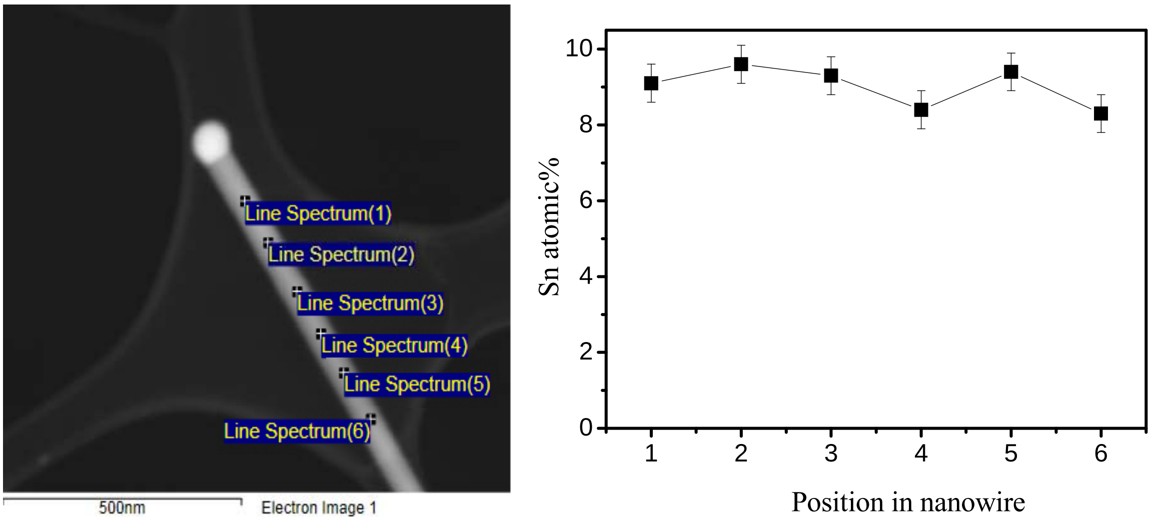

Supplementary Figure 8: **n distribution along length in alloy nanowire with AuAg seed and step cooling.** EDX scan along the length of a nanowire grown with AuAg seed and with an additional step cooling shows uniformity in Sn distribution throughout the nanowire length. Additional annealing step have no ill effect in depositing more Sn near the metallic tip. Error bar represent typical error of 0.5 at.% in EDX measurement.

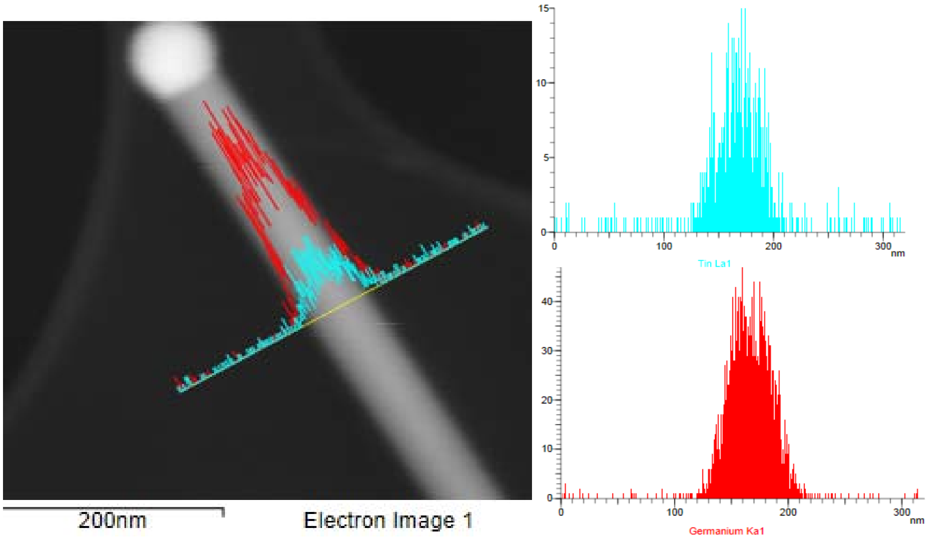

Supplementary Figure 9: **Radial Sn distribution in alloy nanowire with step cooling.** EDX radial concentration profile of Ge and Sn from a nanowire (~ 9 at.% Sn) grown with AuAg catalyst with an additional step cooling. EDX profile shows no segregation of Sn at or near nanowire surface.

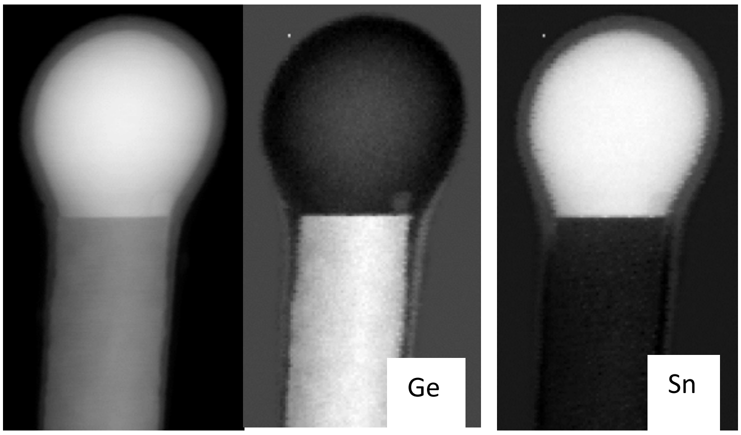

Supplementary Figure 10: **EELS mapping of alloy nanowire**. Low resolution EELS mapping of $Ge_{1-x}Sn_x$ nanowire showing sparse distribution of Sn in the nanowire and high Sn rich catalyst seed.

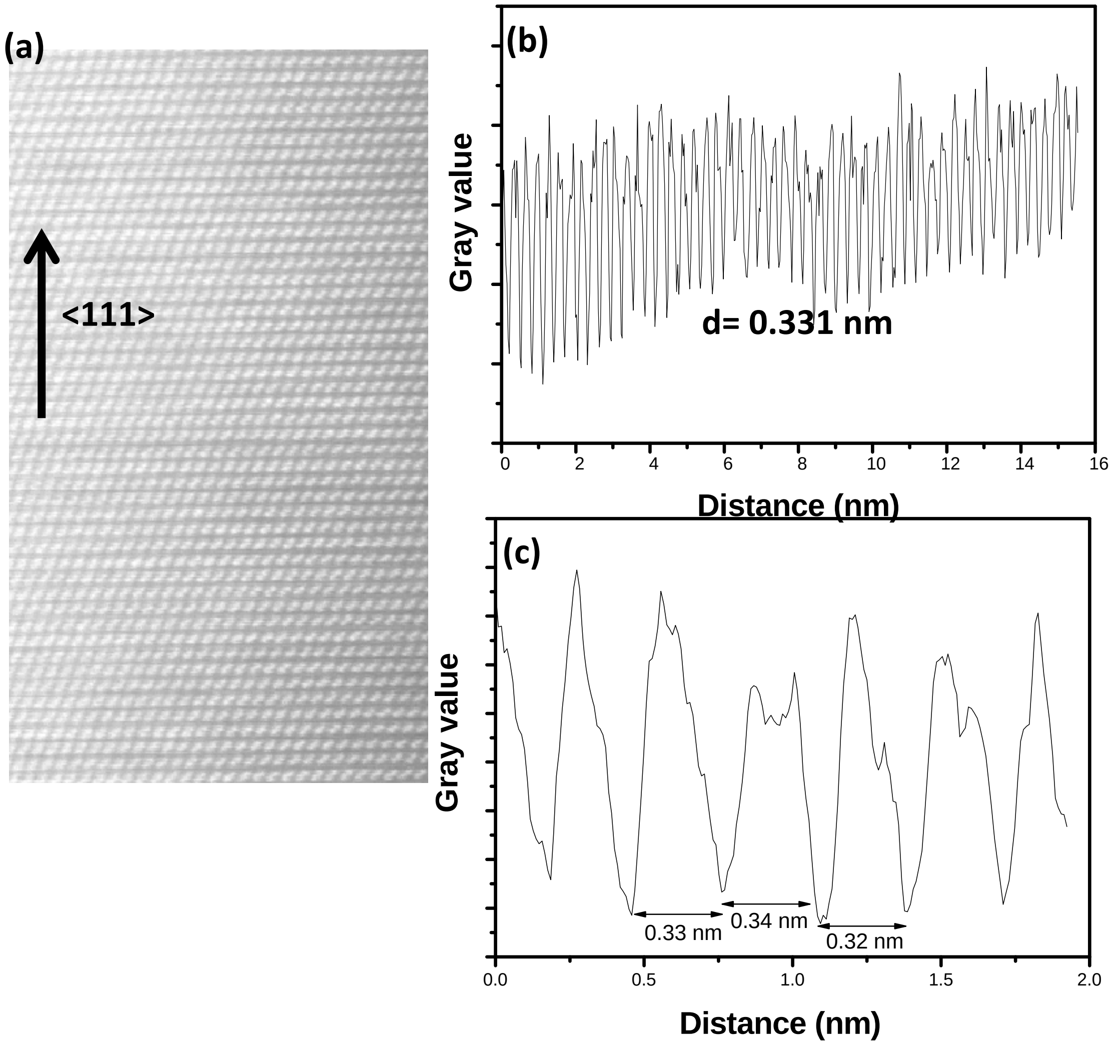

Supplementary Figure 11: **Inter-planar spacing in alloy nanowire.** Calculation of average interplanar spacing from brightness-contrast plot profile from the STEM image in part (a). Average interplanar spacing was calculated to be 3.31 Å from the profile of 50 successive planes (b). Local randomness in the interplanar spacing is evident from the plot (c) with different interplanar spacing (d value) for subsequent planes.

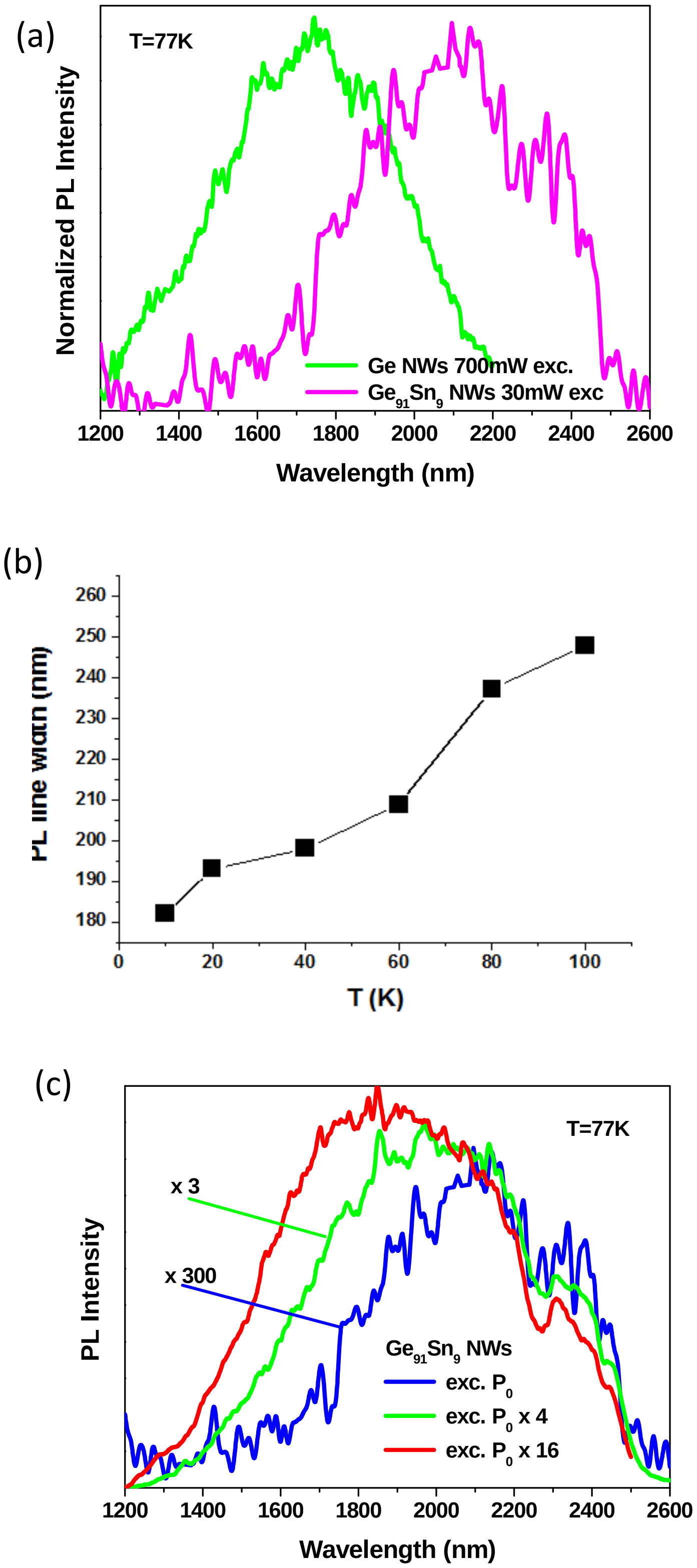

Supplementary Figure 12: Photoluminescence study of alloy nanowire. (a) Comparison between Ge and GeSn nanowires at 77 K. In order to achieve similar emission intensity the excitation power in the case of Ge nanowires was 700 mW in comparison with 30 mW for

GeSn nanowires. (b) PL Line width as a function of temperature for 9.2 at.% Sn incorporated GeSn nanowire. (c) Power-dependent photoluminescence of GeSn nanowires at 77 K. Spectrum for low power (P_0 = 30 mW) enhanced by a factor of 300 for clarity.

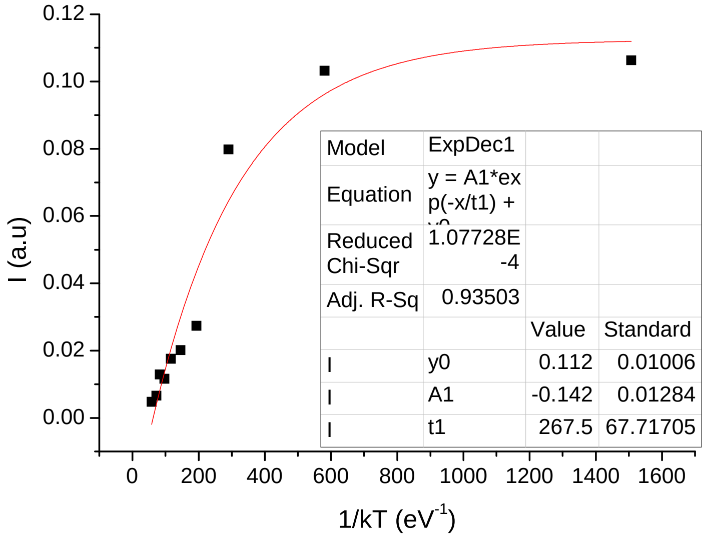

Supplementary Figure 13: Single exponential decay fitting of PL intensity. Arrhenius plot for GeSn nanowires with 6 at.% Sn shows poor agreement with single exponential decay model.

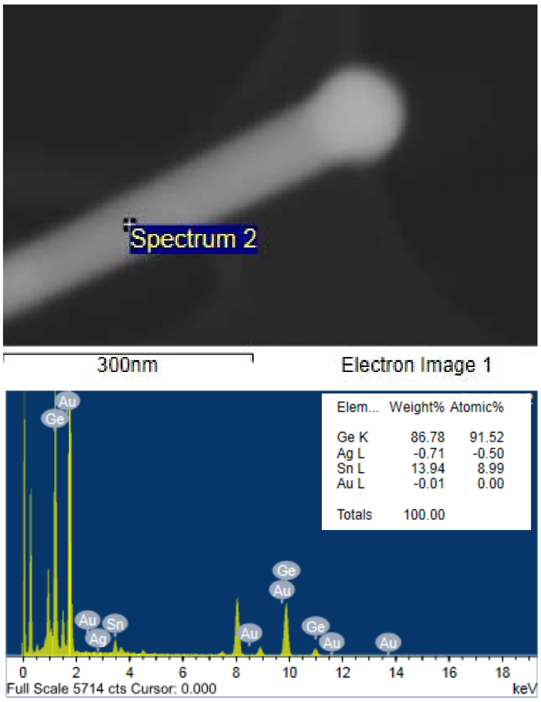

Supplementary Figure 14: **Determination of Au and Ag impurity in nanowires**. Point EDX from $Ge_{1-x}Sn_x$ nanowire grown with step cooling confirms presence of any traceable amount of Au or Ag in the nanowire.

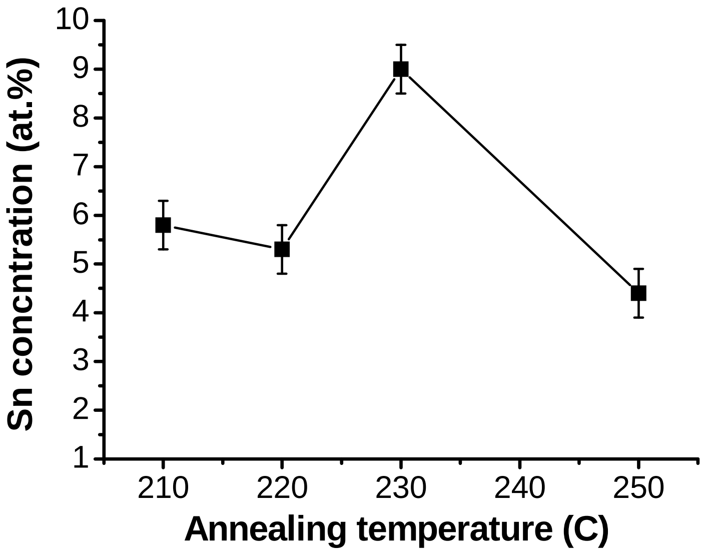

Supplementary Figure 15: **Step cooling at different temperature**. EDX concentration profile of Sn for different nanowires samples with different step cool-down temperature. Highest Sn is observed for 230 ºC. Error bar represent typical error of 0.5 at.% in EDX measurement.
